# Prognostic value and immunological characteristics of a novel cuproptosis-related long noncoding RNAs risk signature in kidney renal clear cell carcinoma

**DOI:** 10.3389/fgene.2022.1009555

**Published:** 2022-11-03

**Authors:** Peng Hong, Weichao Huang, Huifang Du, Ding Hu, Qingfei Cao, Yinjie Wang, Huashan Zhang, Siqiao Tong, Zizhi Li, Ming Tong

**Affiliations:** ^1^ Department of Urology, The First Affiliated Hospital of Jinzhou Medical University, Jinzhou Medical University, Jinzhou, China; ^2^ Department of Radiology, The First Affiliated Hospital of Nanchang University, Nanchang, China; ^3^ The First Clinical College of Jinzhou Medical University, Jinzhou Medical University, Jinzhou, China

**Keywords:** kidney renal clear cell carcinoma, cuproptosis-related lncRNAs, prognostic signature, immunological characteristics, the cancer genome atlas, qRT-PCR

## Abstract

**Background:** Cuproptosis has been found as a novel cell death mode significantly associated with mitochondrial metabolism, which may be significantly associated with the occurrence and growth of tumors. LncRNAs take on critical significance in regulating the development of kidney renal clear cell carcinoma (KIRC), whereas the correlation between cuproptosis-related LncRNAs (CRLs) and KIRC is not clear at present. Therefore, this study built a prognosis signature based on CRLs, which can achieve accurate prediction of the outcome of KIRC patients.

**Methods:** The TCGA database provided the expression profile information and relevant clinical information of KIRC patients. Univariate Cox, Lasso, and multivariate Cox were employed for building a risk signature based on CRLs. Kaplan-Meier (K-M) survival analysis and time-dependent receiver operating characteristic (ROC) curve were employed for the verification and evaluation of the reliability and accuracy of risk signature. Then, qRT-PCR analysis of risk LncRNAs was conducted. Finally, the possible effect of the developed risk signature on the microenvironment for tumor immunization was speculated in accordance with ssGSEA and ESTIMATE algorithms.

**Results:** A prognosis signature composed of *APCDD1L-DT*, *MINCR*, *AL161782.1*, and *AC026401.3* was built based on CRLs. As revealed by the results of the K-M survival study, the OS rate and progression-free survival rate of high_risk_ KIRC patients were lower than those of low_risk_ KIRC patients, and the areas under ROC curves of 1, 3, and 5 years were 0.828, 0.780, and 0.794, separately. The results of the immune analysis showed that there were significant differences in the status of immunization and the microenvironment of tumor between groups at low-risk and at high-risk. The qRT-PCR results showed that the relative expression level of *MINCR* and *APCDD1L-DT* were higher in 786-O and 769-P tumor cells than in HK-2 cells, which were normal renal tubular epithelial cells.

**Conclusion:** The developed risk signature takes on critical significance in the prediction of the prognosis of patients with KIRC, and it can bring a novel direction for immunotherapy and clinical drug treatment of KIRC. In addition, 4 identified risk LncRNAs (especially *APCDD1L-DT* and *MINCR*) can be novel targets for immunotherapy of KIRC patients.

## Introduction

Renal cell carcinoma (RCC), one of the highly common malignant tumor in urology, takes up approximately 3% of all cancers (Umberto et al., 2019; Zachary et al., 2019). Kidney renal clear cell carcinoma (KIRC) is the most common histological type, taking up nearly 80% of RCC (Umberto and Francesco, 2016). In accordance with the U.S. Cancer Statistics 2022, there will be about 79,000 newly diagnosed renal cancer patients and about 13,920 dead renal cancer patients in 2022 years (Siegel et al., 2022). Surgical resection of diseased kidney tissue is still the main treatment for early KIRC, whereas nearly 30% of patients still have recurrence and metastasis after surgical treatment, thus resulting in poor prognosis for a considerable number of KIRC patients (Steven et al., 2017; Giuseppe et al., 2021; Isabel and Grünwald, 2021). Some new treatment methods have been progressively applied to the clinical treatment of KIRC, including vascular endothelial growth factor tyrosine kinase inhibitors (antiangiogenic agents) and immune-checkpoint inhibitors, thus increasing the rate of survival of patients with advanced KIRC to some extent. However, there are still many reports of KIRC recurrence and progression (Sheng and Rini, 2019; Wenzhong et al., 2021). Most patients with advanced KIRC exhibit high mortality, recurrence rate, as well as metastasis rate. Accordingly, novel biomarkers need to be urgently found to identify high-risk KIRC patients with poor prognosis and to build a risk model to evaluate their prognosis for contributing to the clinical diagnosis and prognosis evaluation of KIRC.

Cell death is a critical step in the development of body (Andreas and Vaux, 2020; Hotchkiss et al., 2009). The body is capable of ensuring a healthy and stable microenvironment by inducing damage, aging, and excess cell death (Andreas and Vaux, 2020). A wide variety of cell death methods have been developed over the past few years, such as apoptosis, autophagy, pyroptosis, ferroptosis, and necroptosis (Daniel and Vince, 2019; Mark, 2019; Stockwell, 2022). Recently, a new process of cell death has been discovered—Cuproptosis, which occurs through the direct combination of copper ions with fatty acyl components in the tricarboxylic acid cycle in mitochondrial respiration, which leads to the aggregation of fatty acyl protein and the following reduction of iron-sulfur cluster proteins, thus resulting in protein toxic stress and eventually cell death (Cobine and Brady, 2022; Daolin et al., 2022; Peter et al., 2022). The mechanism of cuproptosis is not consistent with other known cell death mechanisms. Cell death induced by copper ionophore mainly depends on the accumulation of copper in cells. Cell death induced by copper ionophore is a novel cell death process, obviously inconsistent with the conventional way of cell death. After reviewing the relevant literature, we found that the occurrence of KIRC is usually accompanied by the reprogramming of the tricarboxylic acid cycle. By reducing the energy generated by the tricarboxylic acid cycle, KIRC enables tumor cells to survive under harsh conditions and escape from the surveillance and attack of the immune system (Cancer Genome Atlas Research Network, 2013; Marston et al., 2019). In addition, there have been some reports on the progress of cuproptosis in cancer research, such as cuproptosis-related genes can predict the prognosis and immunotherapy sensitivity of pancreatic cancer patients (Yingkun et al., 2022). We need to study the roles and specific mechanisms of cuproptosis in tumorigenesis and development in depth and find specific biomarkers, which can show novel directions for KIRC diagnosis and treatment.

Long non-coding RNAs (LncRNAs) refer to non-coding RNAs containing the length of over 200 nucleotides. RNA polymerase II transcribe the LncRNAs. In recent years, many studies have reported that LncRNAs take on vital significance in the occurrence and growth of KIRC. For instance, *LncRNA SNHG1* is capable of activating *STAT3* and *PD-L1* as competitive endogenous RNA of *miR-129-3p* (Pei et al., 2021), which can lead to the regulation of the immune escape of renal cell carcinoma. The result of another study suggests that knocking down *LncRNA LINC00944* leads to significantly inhibits the proliferation and migration of renal cell carcinoma and facilitates AKT phosphorylation (Chen and Zheng, 2021). *LncRNA GAPLINC* can promote the tumorigenesis of renal cell carcinoma by targeting *miR-135b-5p/CSF1* axis (Wang et al., 2021). In addition, many LncRNAs-related prognostic models have been reported in KIRC. For instance, the m7G-related LncRNAs prognostic model can accurately achieve the prediction of the prognosis of KIRC patients (Jie and Chunyang, 2022), immune-associated LncRNAs prognosis signature has prognostic significance in KIRC (Seema et al., 2019), and ferroptosis-related LncRNAs can provide accurate prognosis prediction for KIRC patients (Xiao-Liang et al., 2021). However, cuproptosis is a novel cell death mode, and there has been little research about the Cuproptosis-related LncRNAs (CRLs) prognosis model in KIRC.

In this study, based on CRLs, our team built and verified a risk signature for evaluating and improving the prognosis of KIRC patients, and verified its clinical value. In addition, it shows the feasibility that the risk signature can make personalized immunotherapy and targeted therapy for KIRC patients.

## Materials and methods

### Data sources

**FIGURE 1:**
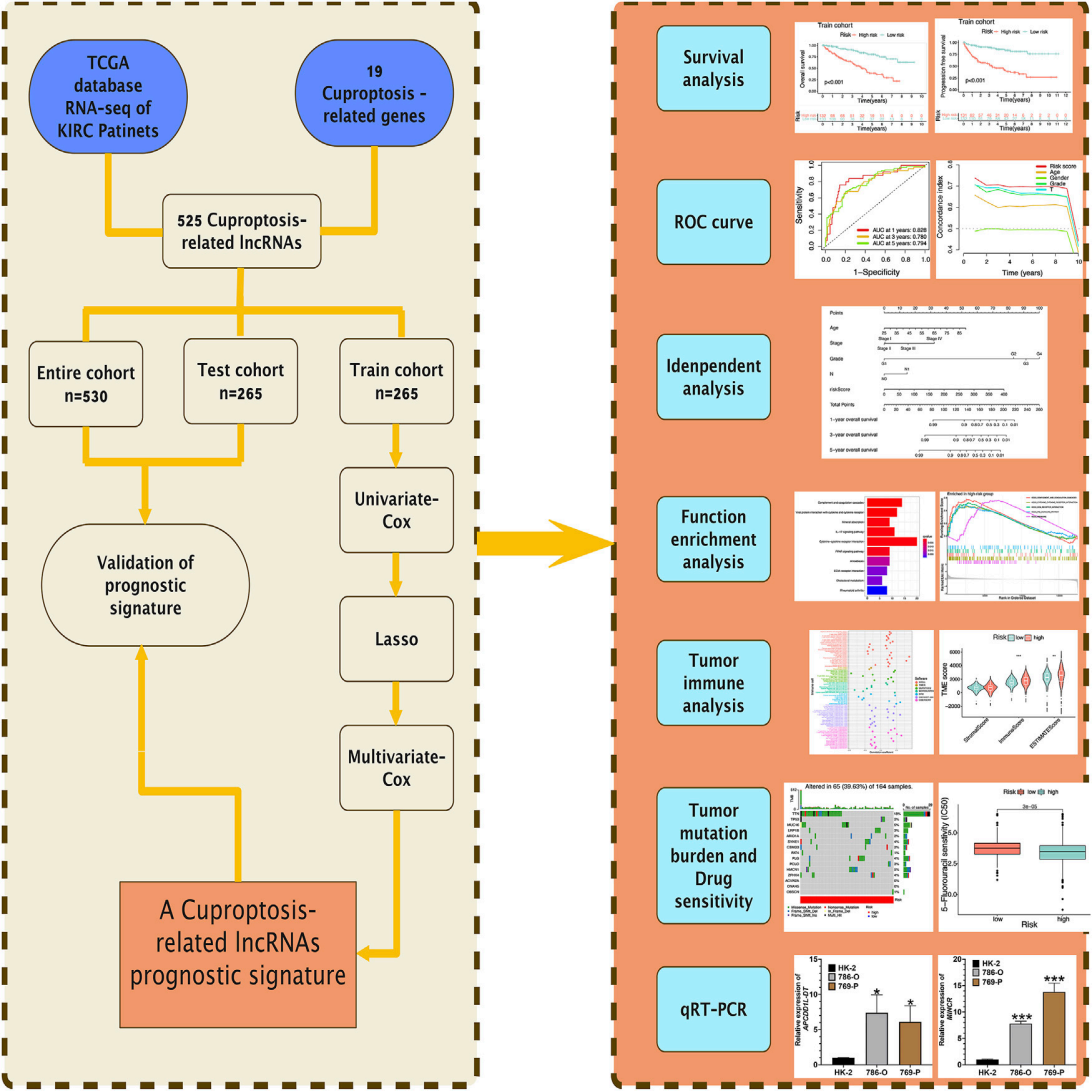
The research steps of this article.

Figure 1 presents the flow of this study. The Cancer Genome Atlas (TCGA) database (https://portal.gdc.cancer.gov) is a database jointly developed by the National Cancer Institute and the National Human Genome Research Institute, which contains clinical data, genomic variation, mRNA expression, miRNA expression, methylation, and other data of various human cancers. TCGA database provided 72 healthy renal tissular specimens as well as RNA sequencing data of 539 tissular specimens of KIRC in the format of “HTSEQ-FPKM, TCGA-KIRC” (Marston and J, 2019). Furthermore, the TCGA database provided relevant clinical information. We employed 530 KIRC samples containing complete RNA sequencing information and clinical information for the following analysis by excluding several samples containing not complete data. The 530 KIRC samples were randomized 1:1 as the group of testing (n = 265, to verify the CRLs risk signature) as well as the group of training (n = 265, to develop the CRLs risk signature) (Table 1).

**TABLE 1:** Clinical information of KIRC patients in Train cohort, Test cohort, and Entire TCGA cohort.

Covariates	Type	Entire TCGA cohort	Test cohort	Train cohort	*p*value
Age	≤65	348 (65.66%)	174 (65.66%)	174 (65.66%)	1
	>65	182 (34.34%)	91 (34.34%)	91 (34.34%)	
Gender	FEMALE	186 (35.09%)	87 (32.83%)	99 (37.36%)	0.3168
	MALE	344 (64.91%)	178 (67.17%)	166 (62.64%)	
Grade	G1	14 (2.64%)	5 (1.89%)	9 (3.4%)	0.7295
	G2	227 (42.83%)	112 (42.26%)	115 (43.4%)	
	G3	206 (38.87%)	103 (38.87%)	103 (38.87%)	
	G4	75 (14.15%)	41 (15.47%)	34 (12.83%)	
	GX	5 (0.94%)	3 (1.13%)	2 (0.75%)	
	unknow	3 (0.57%)	1 (0.38%)	2 (0.75%)	
Stage	Stage I	265 (50%)	137 (51.7%)	128 (48.3%)	0.3438
	Stage II	57 (10.75%)	24 (9.06%)	33 (12.45%)	
	Stage III	123 (23.21%)	65 (24.53%)	58 (21.89%)	
	Stage IV	82 (15.47%)	36 (13.58%)	46 (17.36%)	
	unknow	3 (0.57%)	3 (1.13%)	0 (0%)	
T	T1	21 (3.96%)	14 (5.28%)	7 (2.64%)	0.7918
	T1a	140 (26.42%)	67 (25.28%)	73 (27.55%)	
	T1b	110 (20.75%)	59 (22.26%)	51 (19.25%)	
	T2	55 (10.38%)	22 (8.3%)	33 (12.45%)	
	T2a	10 (1.89%)	4 (1.51%)	6 (2.26%)	
	T2b	4 (0.75%)	2 (0.75%)	2 (0.75%)	
	T3	5 (0.94%)	2 (0.75%)	3 (1.13%)	
	T3a	120 (22.64%)	62 (23.4%)	58 (21.89%)	
	T3b	52 (9.81%)	27 (10.19%)	25 (9.43%)	
	T3c	2 (0.38%)	1 (0.38%)	1 (0.38%)	
	T4	11 (2.08%)	5 (1.89%)	6 (2.26%)	
M	M0	420 (79.25%)	216 (81.51%)	204 (76.98%)	0.5249
	M1	78 (14.72%)	35 (13.21%)	43 (16.23%)	
	MX	30 (5.66%)	14 (5.28%)	16 (6.04%)	
	unknow	2 (0.38%)	0 (0%)	2 (0.75%)	
N	N0	239 (45.09%)	125 (47.17%)	114 (43.02%)	0.5913
	N1	16 (3.02%)	7 (2.64%)	9 (3.4%)	
	NX	275 (51.89%)	133 (50.19%)	142 (53.58%)	

UCSC Xena (https://xenabrowser.net/) offered copy number variation (CNV) data and tumor mutation data for KIRC patients. Choose the “KIRC.Varscan. Somatic. maf.” file for subsequent tumor mutation burden (TMB) analysis. Moreover, tumor immune dysfunction and exclusion (TIDE) score data for each KIRC patient were obtained from the TIDE website (http://tide.dfci.harvard.edu). In accordance with previous studies and literature reports, we obtained a total of 19 cuproptosis-related genes (*DLSTGCSH, DBT, CDKN2A, GLS, MTF1, PDHB, PDHA1, DLAT, DLD, LIPT2, LIPT1, LIAS, FDX1, SLC31A1, ATP7A, ATP7B, NLRP3, NFE2L2*) (Ok et al., 2014; Francesco and Serena, 2017; Anna et al., 2018; Ze et al., 2019; Jianjian et al., 2021; Peter et al., 2022)*.* The above data were all from public databases, which ensured the reproducibility of the study.

### Expression and mutation analysis of cuproptosis-related genes

The expression differences of cuproptosis-related genes in healthy renal tissular specimens and KIRC tissular specimens were analyzed by R package “limma” in R program, and corresponding boxplots were plotted by R package “ggpubr”. Mutations in cuproptosis-related genes were represented by a waterfall plot by R Package “maltools”. The CNV frequency of cuproptosis-related genes was shown in the bar chart.

### Identification of cuproptosis-related LncRNAs

TCGA-KIRC transcriptome data were divided into mRNA and LncRNA using Perl script based on GTF files (human transcriptome annotates information). The correlation between cuproptosis-related genes and LncRNAs expression was analyzed using R package “limma” through Pearson correlation analysis, with **|**correlation coefficient**|** >0.4, *p* < 0.001 as the filter criterion to obtain CRLs.

### Establishment and validation of prediction signature based on cuproptosis-related LncRNAs

The Train cohort was used for the development of the risk signature, and the Test cohort as well as the Entire TCGA cohort were employed to verify the built risk signature. Based on the overall survival (OS) time in clinical information of patients of KIRC, univariate Cox analysis was employed to evaluate the prognosis significance of CRLs (False Discovery Rate (FDR) < 0.05). In order to prevent over-fitting in the development of the risk signature, R package “glment” was employed to further optimize the selection of prognostic CRLs using Lasso regression analysis. We carried out multivariate Cox regression analysis for the above most representative prognostic CRLs to obtain the hazard ratio (HR) and regression coefficients for the respective risk LncRNA. Based on the mentioned investigation, detailed risk LncRNAs and the regression coefficients were presented, and risk signatures based on CRLs were built. The risk score of the respective KIRC patient was obtained as follows:

$$risk\;score=\sum_{i=1}^{n}coef\left(LncRNA\right)*\mathrm{Exp}\left(LncRNA\right)$$

All KIRC patients were classified into two groups, including risk_high_ and risk_low_ according to the median risk score of the Train cohort as the cutoff. R-package “survival” and “survminer” were adopted to analyze whether OS and progression-free survival (PFS) of KIRC patients are different between the two risk groups through Kaplan-Meier (K-M) survival study. R-package “survival”, “survminer” and “timeROC” were employed to generate multiple receiver operating characteristic (ROC) curves. We obtained the area under the ROC curve (AUC) for verifying the predictive value of the prognostic signature and evaluating the accuracy of the risk signature in the prediction of KIRC patient’s prognosis. In addition, we adopted the concordance index for evaluating the prediction accuracy of the risk signature. Lastly, univariate Cox and multivariate Cox regression analyses were used for the investigation of whether the risk signatures or other clinical characteristics may be the independent prognostic indicators of KIRC patients. The results of regression analysis were presented in forest maps. The above analyses were validated in both the Test cohort and the Entire TCGA cohort.

### Establishment and calibration of nomogram

A model for the identification of nomogram risk was developed with the use of R-package “rms” based on some independent factors for prognosis in the clinical field and risk scores. Nomogram is capable of quantifying the factor for KIRC patient’s prognosis and carry out the quantitative prediction of KIRC patients’ prognosis. Next, we generated calibration curves for illustrating the built nomogram’s prediction effect. The calibration of the respective model was presented by the above curves in accordance with the condition that the actual time of survival of KIRC patients was consistent with the estimated time of survival of KIRC patients. The y-axis represents the actual time of survival of KIRC patients. The estimated time of survival of KIRC patients was represented by the x-axis. The perfectly predicted model for risk was represented by the light grey line. Better prediction was represented by the light grey line closer to the diagonal, and the nomogram performance is represented by the pink solid line.

### Functional enrichment study

R package “limma” (screening criteria: |log2Fold Change (FC)| > 1, FDR <0.05) was adopted to screen genes with differential expression in risk_high_ and risk_low_ groups. Subsequently, Gene Ontology (GO) and Kyoto Encyclopedia of Genes and Genomes (KEGG) enrichment analysis were performed for the above genes (R package “clusterProfiler”). Gene Set Enrichment Analysis (GSEA) was conducted based on the gene set files of “c2.cp.kegg.v7.4.symbols.gmt”, R package “limma”, “clusterProfiler”, “org.Hs.eg.db” and “enrichplot” were employed to identify significantly enriched pathways in risk_high_ and risk_low_ KIRC patients (|log2FC| > 1, FDR <0.05), respectively.

### Analysis of tumor immune microenvironment

The situation of a wide variety of immune cell infiltration in different KIRC samples was obtained using seven 7 algorithms (CIBERSORT-ABS, CIBERSORT, EPIC, MCPCOUNTER, QUANTISEQ, TIMER, XCELL). Next, the Spearman correlation study was used for investigating the correlation between different immune cell infiltration degrees and risk scores. The above algorithms were systematically benchmarked, and each of them exhibited unique performance and advantages. Then, the content of immunocytes in the respective KIRC sample was quantified using the algorithm of “Cell-type Identification based on the Estimation of Relative Subsets of RNA Transcripts (CIBERSORT)” (Newman et al., 2015; Binbin et al., 2018). The single sample Gene Set Enrichment Analysis (ssGSEA) was used for the analysis of differences in the score of enrichment of 13 immunization-associated pathways and 16 immunocytes in different risk groups, the analysis results were presented in multi-box diagram. The StromalScore, ImmuneScore, and EstimateScore (StromalScore + ImmuneScore) were obtained using the algorithm of “Estimating Stromal and Immunocytes in MAlignant Tumor tissues based on Expression data (ESTIMATE)” (Kosuke et al., 2013) for the respective KIRC patient and then for the comparison of the score differences of a wide variety of risk groups. Moreover, the comparison of the expression differences for some common immune-checkpoints genes in different risk groups and then used the TIDE algorithm to predict potential immunotherapy responses. Based on the immunosuppressive factors (including short survival after ICB treatment, poor efficacy of immune checkpoint blocking therapy (ICB), as well as high TIDE score), TIDE evaluated two mechanisms of tumor immune escape (rejection of cytotoxic T lymphocyte and tumor-infiltrating CTL dysfunction) by employing several markers of gene expression (Peng et al., 2018).

### Prediction of potential drug sensitivity

Based on R package “pRRophetic”, the prediction was conducted, the sample’s maximum 50% inhibition concentration (IC50) was predicted using ridge regression, and IC50 represented 50% of the suppressed cells, i.e., the cell survival rate was half of the control sample. In other words, the lower the IC50 value of the corresponding drug concentration, the more sensitive KIRC patients will be to the drug (Paul et al., 2014).

### Cell culture

In the First Affiliated Hospital of Jinzhou Medical University, the human KIRC cell lines 786-O and 769-P used in this study were preserved. Procell Company in Wuhan, China provided normal renal tubular epithelial cell line HK-2. RPMI-1640 medium (Hyclone) achieved the culture of 768-O and 769-P cell lines, while MEM medium (Hyclone) achieved the culture of HK-2 cells, with the addition of 10% fetal bovine serum (FBS) and 1% penicillin/streptomycin, respectively. All cells were incubated at 37°C containing 95% air and 5% carbon dioxide.

### RNA extraction and quantitative real-time polymerase chain reaction

In accordance with cultured 786-O, 769-P, and HK-2 cells, total RNA was obtained by TRIzol (Beyotime, Shanghai, China), and then reverse transcribed into cDNA by reverse transcription kit (Beyotime, Shanghai, China) on PCR Cycler (Bio-Rad, United States). We employed SYBR-Green mixture (Beyotime, Shanghai, China) and Bio-Rad chemiluminescence imager (Bio-Rad, United States) for qRT-PCR. All the above experimental steps were conducted in accordance with the product instructions, and the amplified primer sequences were as follows:

*APCDD1L-DT*, forward primer: GAG​CCT​TGG​AAA​GGA​GGA​CC, reverse primer: GAT​CCA​TGC​AGG​TGG​GAA​CA.
*MINCR*, forward primer: TCC​AAG​GTC​GAT​TTT​CTT​AGC​CA, reverse primer: CCC​TTT​TCA​GTT​CAC​AAG​CGT.
*GAPDH*, forward primer: TCG​TGG​AAG​GAC​TCA​TGA​CC, reverse primer: TCC​ACC​ACC​CTG​TTG​CTG​TA.

*GAPDH* served as internal control, the relative expression was examined by 2^^−ΔΔCt^. The experiment was repeated three times.

### Statistical analysis

To conduct the K-M survival analysis for generating the survival curve, we performed Log-rank test. For examining the diversities between a variety of classified data or different datasets, we carried out the Chi-square test. For determining the difference between the above two groups, we carried out Wilcoxon-rank test. For the analysis of correlation, we employed Spearman method. For the assessment of the effect exerted by gene expression, clinical features, and risk signature on patients’ prognosis, we carried out cox proportional regressive analysis. The above statistical methods achieved statistical significance if *p* < 0.05. The analysis was performed based on R version 4.1.0 and the feature package.

## Results

### Biological characterization of 19 cuproptosis-related genes in KIRC

**FIGURE 2:**
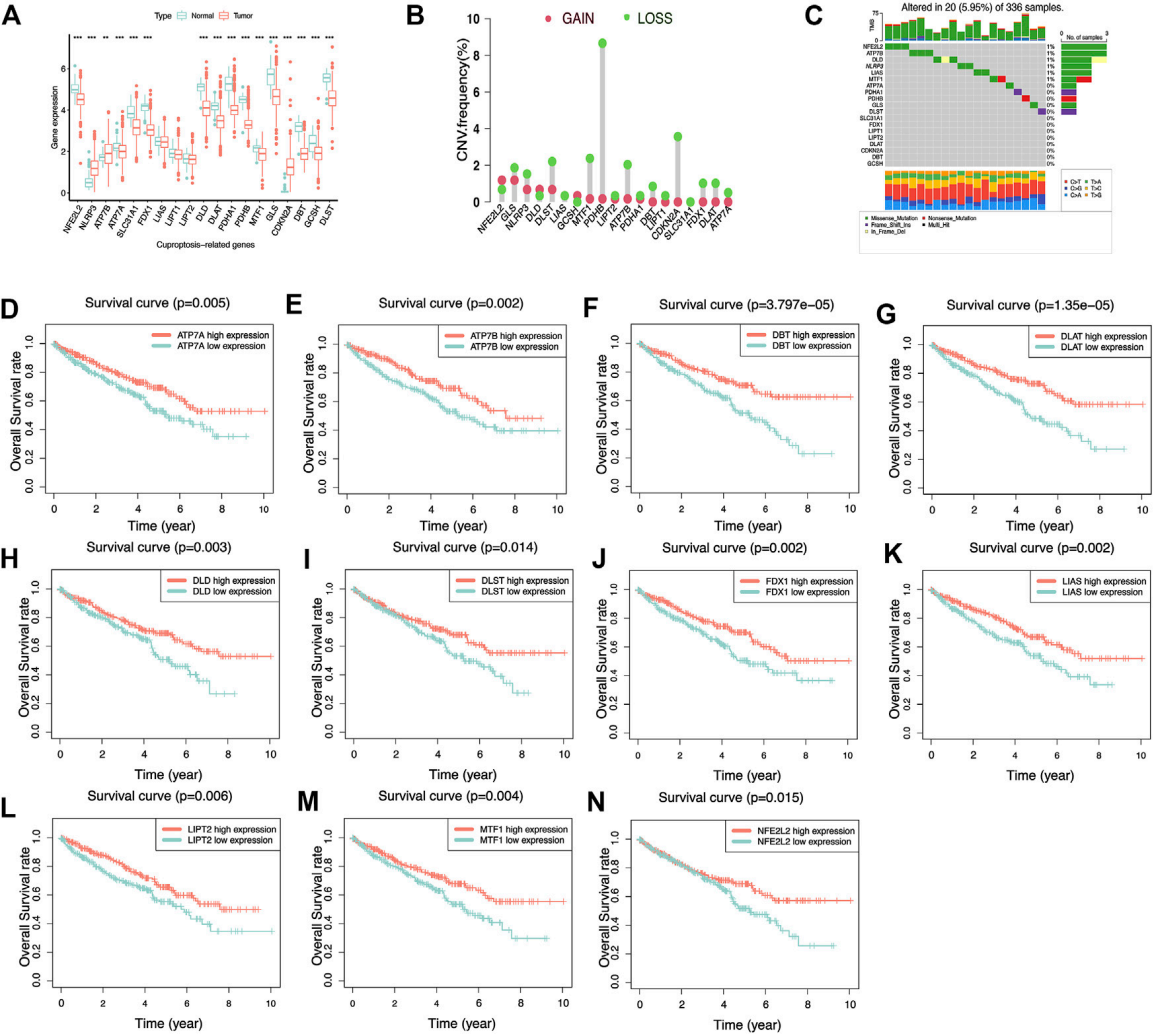
Biological characteristics of 19 cuproptosis-related genes. **(A)** Differential expression of cuproptosis-related genes in KIRC and normal renal tissues. ** *p*< 0.01, *** *p*< 0.001. **(B)** Copy number variation frequency plots showed that most cuproptosis-related genes had copy number deletions. **(C)** The waterfall plot shows the frequency of cuproptosis-related genes mutations in KIRC. The different colored squares at the bottom represent different types of mutations. **(D–N)** K–M survival curves of cuproptosis-related genes in KIRC. Most of the cuproptosis-related genes in KIRC have prognostic significance and act as an adverse prognostic factor.

First, We extracted the expression data of 19 genes associated with cuproptosis from 539 KIRC tissue specimens and 72 normal kidney tissue specimens and further analyzed the expression of the above 19 genes associated with cuproptosis in KIRC tissue specimens and normal kidney tissue specimens. It was found that the expression of most genes associated with cuproptosis was lower expression in KIRC tumor tissue, compared with that in normal kidney tissue specimens (Figure 2A). We analyzed the 19 genes associated with cuproptosis CNV and somatic mutations in KIRC, at the level of CNV, we found that most of the genes associated with cuproptosis were focused on the loss of copy number (Figure 2B). In 336 KIRC specimens, there were 20 specimens carry genes associated with cuproptosis mutations, and the *NFE2L2* mutation frequency was the highest (Figure 2C). It is speculated that CNV differences and genetic mutations may mediate the difference in expression of genes associated with cuproptosis between normal kidney tissue and KIRC tissue. Subsequently, the influence of the above genes associated with cuproptosis on the OS rate of KIRC patients was analyzed, and it was also surprising to find that most of the genes associated with cuproptosis were significantly associated with the survival of KIRC (Figure 2D–N). The above analyses suggested that the imbalance of the expression of genes associated with cuproptosis may affect the occurrence and growth of KIRC patients.

### Establishment and validation of prediction signature based on LncRNAs associated with cuproptosis

**FIGURE 3:**
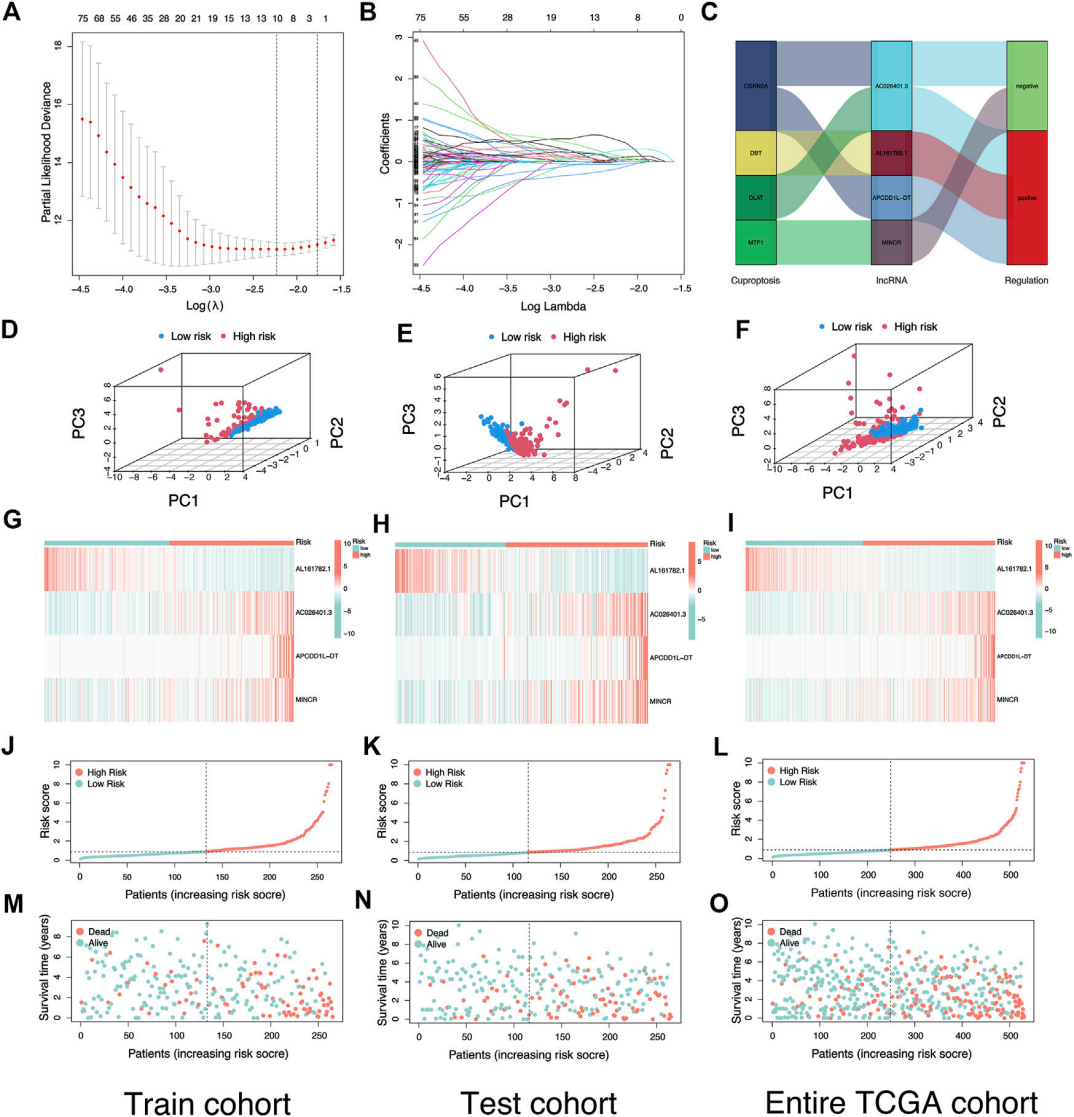
Construction and verification of risk signature based on cuproptosis-related lncRNAs. **(A)** Cross-validation for variable selection in the LASSO model. **(B)** Lasso coefficient distribution of risk cuproptosis-related lncRNAs. **(C)** Sankey diagram illustrates the regulatory relationship between Cuproptosis-related genes and lncRNAs. **(D–F)** The Principal Component Analysis in Train cohort, Test cohort, and Entire TCGA cohort based on different risk groups. **(G–I)** Heatmap of the expression of four risk lncRNAs in different risk groups in Train cohort, Test cohort, and Entire TCGA cohort. **(J–L)** Risk plot distribution of Train cohort, Test cohort, and Entire TCGA cohort. **(M–O)** Distribution of survival status of KIRC patients in Train cohort, Test cohort, and Entire TCGA cohort.

A total of 13349 LncRNAs expression data were obtained from TCGA-KIRC transcriptome data. Subsequently, we carried out Pearson correlation study in accordance with the expression of 19 genes associated with cuproptosis and LncRNAs expression data. A total of 525 LncRNAs were consistent with the corresponding conditions, that was, |correlation coefficient| > 0.4, *p* < 0.001, and were defined as CRLs (Supplementary Files S1). In the Train cohort, 525 CRLs were analyzed by univariate Cox regression analysis, and 192 CRLs with prognostic values were obtained. Next, we performed LASSO regression analysis on 192 prognostic CRLs for eliminating highly correlated prognostic LncRNAs and avoiding overfitting (Figure 3A,B) for the optimization of the developed signatures. We found the 10 most representative CRLs. Cross-validation results showed that LASSO regression analysis was the best. Multivariate Cox regression analysis was performed for the 10 most representative prognostic CRLs to obtain the hazard ratio (HR) and regression coefficients for 4 risk LncRNAs. The risk score for each KIRC patient can be obtained as:

$$\begin{array}{l} Risk\;score=\left(-1.15122394874834*{\mathrm{Exp}}_{AL161782.1}\right)+\left(0.4711103719724987*{\mathrm{Exp}}_{AC026401.3}\right)+\left(0.678892201655986*{\mathrm{Exp}}_{APCDDIL-DT}\right)\\ +\left(0.468667562066302*{\mathrm{Exp}}_{MINCR}\right) \end{array}$$

**FIGURE 4:**
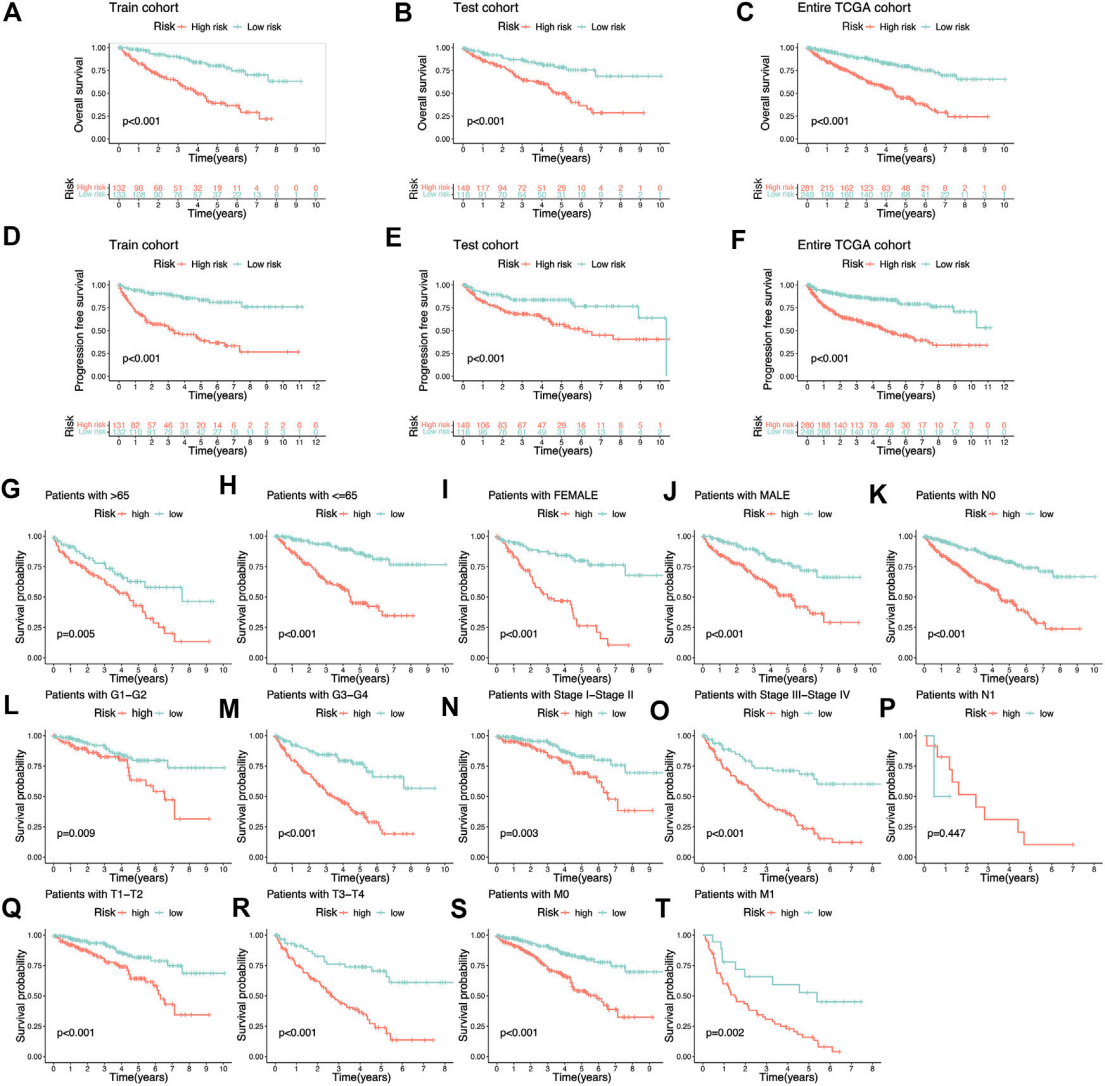
K-M survival analysis based on cuproptosis-related lncRNAs risk signature. **(A–C)** The Overall survival K-M survival curves of different risk groups in Train cohort, Test cohort, and Entire TCGA cohort. The overall survival of patients with high-risk KIRC is lower than that of patients with low-risk KIRC. **(D–F)** The Progression-free survival K-M survival curves of different risk groups in Train cohort, Test cohort, and Entire TCGA cohort. The Progression-free survival of patients with high-risk KIRC is lower than that of patients with low-risk KIRC. **(G–T)** The Overall survival K-M survival curves of different risk groups with different clinical features.

**FIGURE 5:**
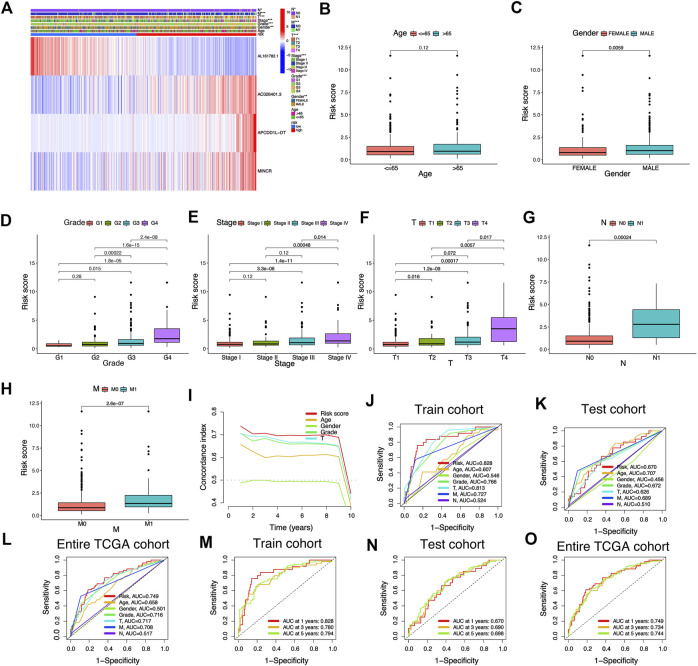
Association between risk signature scores composed of cuproptosis-related lncRNAs and clinical features. **(A)** The heatmap shows the clinical characteristics and expression of 4 risk cuproptosis-related lncRNAs in 530 KIRC patients. **(B–H)** Differential distribution of risk scores among KIRC patients with different clinical characteristics. **(I)** The consistency index showed that the risk score was better than other clinicopathological features in predicting the prognosis of KIRC patients. **(J–O)** ROC curves for risk signature composed of cuproptosis-related lncRNAs in Train cohort, Test cohort, and Entire TCGA cohort.

The Sankey diagram showed the regulatory relationship between 4 risk LncRNAs and genes associated with cuproptosis (Figure 3C). All KIRC patients were divided into risk_high_ and risk_low_ groups in accordance with the median risk score in the Train cohort as a critical point. Principal component analysis (PCA) showed that risk scores could significantly divide KIRC patients into risk_high_ and risk_low_ groups (Figures 3D–F). We also compared the expression of the above 4 risk LncRNAs in the risk_high_ and risk_low_ groups (Figure 3G–I). Subsequently, we rank the survival state and risk score distribution of patients with KIRC, and we could see that the number of deaths of patients with KIRC was increasing with the increase of risk score (Figure 3J–O). In accordance with the K-M survival analysis, we also found that the OS rate of the risk_high_ group was lower than that of the risk_low_ group (Figure 4A–C). In addition, a significant difference was identified in PFS rate between the risk_high_ and risk_low_ groups, that is, the PFS rate of the risk_high_ group was lower than that of the risk_low_ group (Figure 4D–F). The same survival analysis results were obtained for KIRC patients with different clinical characteristics (except stage N1 KIRC patients) (Figure 4G–T). The explanation for no significant difference in OS rate between risk_high_ and risk_low_ groups of KIRC patients in N1 may be that there were fewer KIRC patients in N1, revealing that the developed risk signature is applicable to KIRC patients exhibiting nearly all clinical characteristics. The heatmap showed each KIRC patient’s clinical characteristics and risk scores (Figure 5A). As depicted in the figure, there were significant differences in the risk scores of KIRC patients with different clinical characteristics. To be specific, the risk scores of KIRC patients in M1, N1, G3-G4, and T3-T4 were higher than those of KIRC patients in MO, N0, G1-G2, and T1-T2, respectively (Figure 5B–H). In general, the risk scores of patients of KIRC with advanced was often higher.

### Assessment of risk signature

We adopted the area under ROC curve for evaluating the accuracy of risk signature in the prediction of the outcome of patients with KIRC. The results showed that the areas under ROC curve of the Train cohort 1, 3, and 5 years were 0.828, 0.780, and 0.794 respectively (Figure 5J); the areas under ROC curve of the Test cohort 1, 3, and 5 years were 0.670, 0.690 and 0.698 respectively (Figure 5K); the areas under ROC curve of the Entire TCGA cohort 1, 3 and 5 years were: 0.749, 0.734 and 0.744 respectively (Figure 5L). In addition, we also found that the risk score was better than other clinical variables in the prediction of the prognosis of patients with KIRC (Figure 5M–O). The concordance index also confirmed that the risk score was more accurate in predicting KIRC patients’ outcomes than other clinicopathological features (Figure 5I). The above results demonstrated the ability of our risk signature to accurately achieve the prediction of the prognosis of patients with KIRC.

### Risk signature have excellent independent prognostic value

**FIGURE 6:**
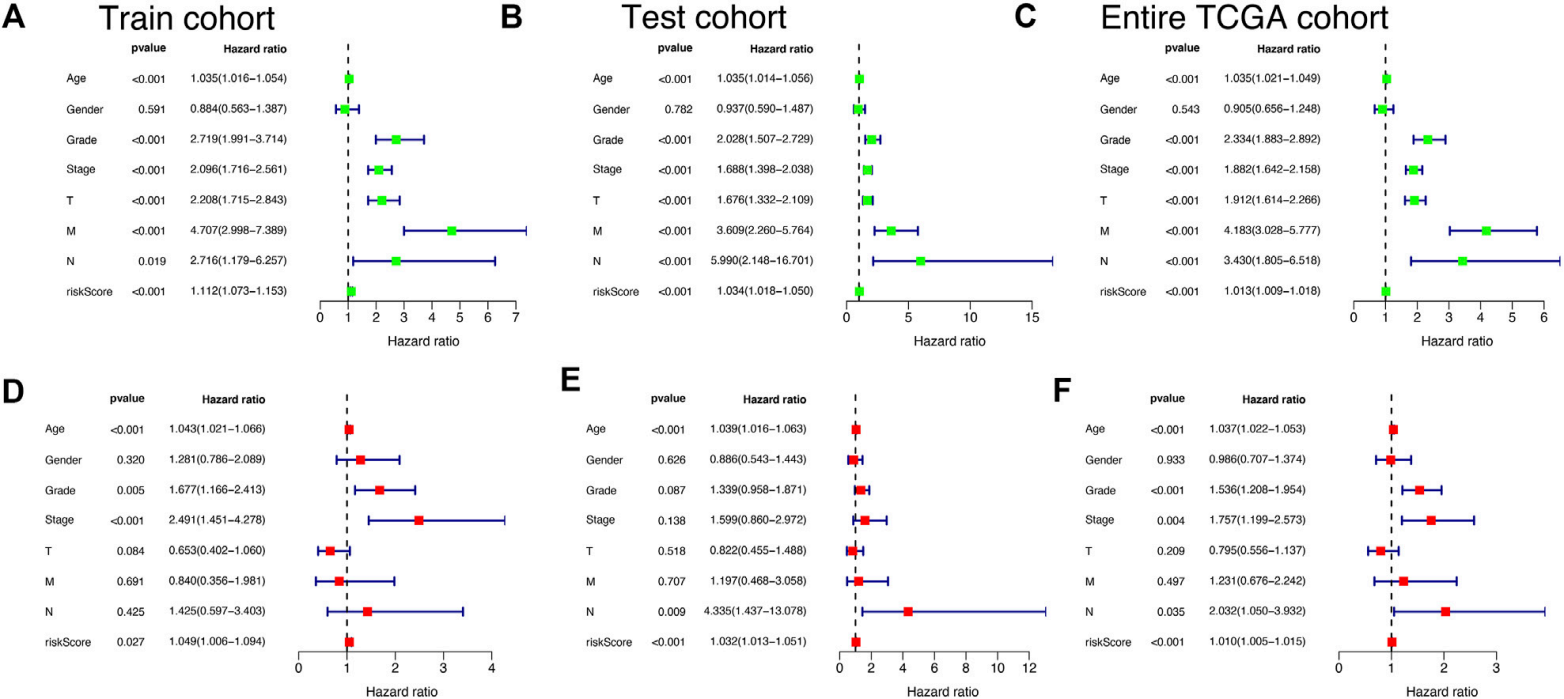
Independent prognostic validation of risk signatures. Univariate and multivariate Cox regression analysis results were presented in the form of forest plots, indicating that risk signature was an independent prognostic factor for KIRC patients in Train cohort **(A and D)**, Test cohort **(B and E)**, and Entire TCGA cohort **(C and F)**.

Univariate-multivariate Cox regression analysis was performed to determine whether the risk score was an independent predictor of patients’ outcome, regardless of other clinical characteristics. In the Train cohort, univariate Cox results showed that risk score was significantly associated with patient prognosis (Figure 6A). Multivariate Cox regression results further demonstrated that risk score can be used as a biomarker of independent prognosis for KIRC patients regardless of clinical characteristics (Figure 6D). The same uni-multi Cox regression analysis results were obtained for the Test cohort and the Entire TCGA cohort (Figure 6B,C,E,F). The above results suggest that the risk signature built based on CRLs has certain significance for the prognostic assessment of KIRC patients.

### Nomogram

**FIGURE 7:**
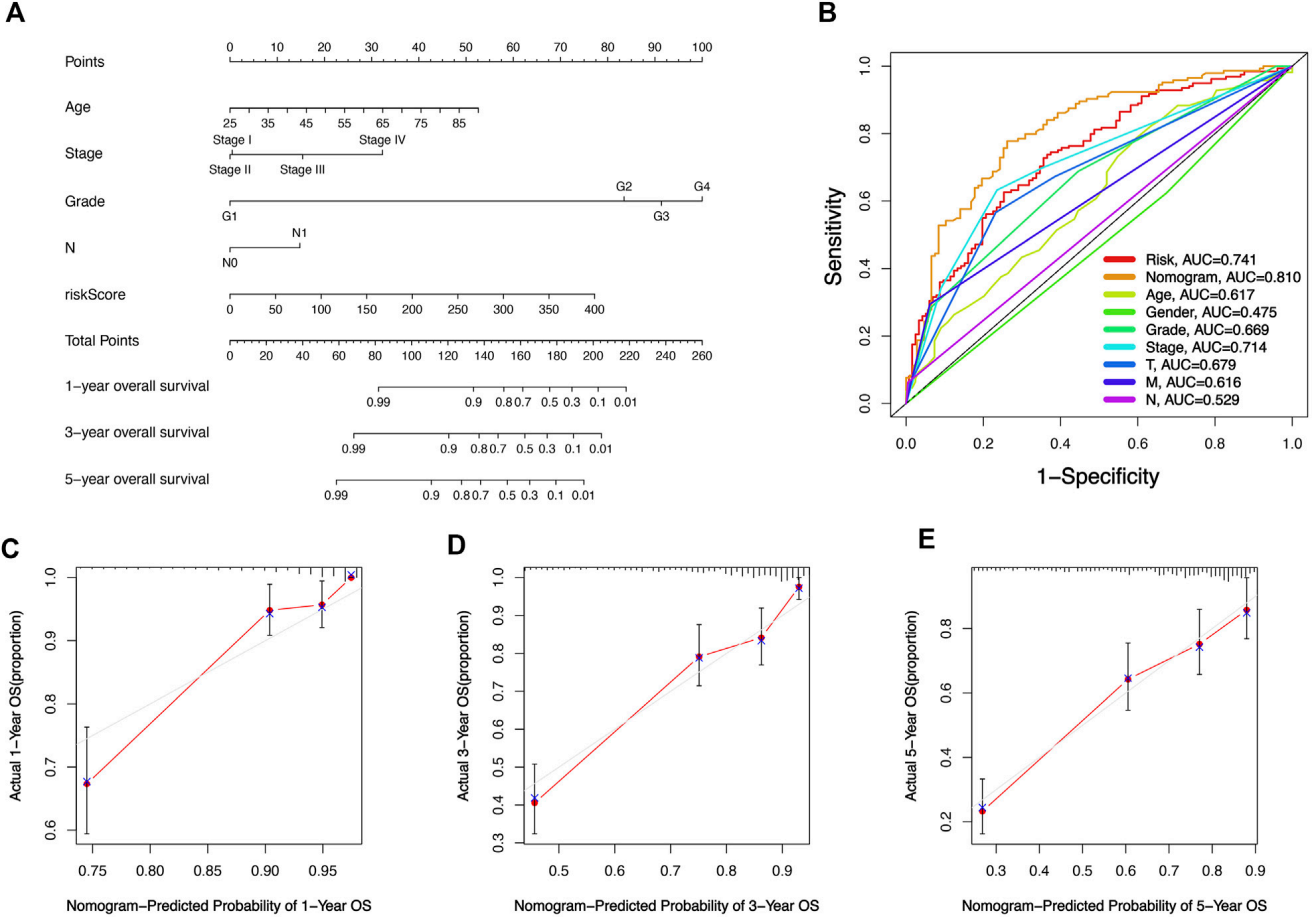
Establishment and validation of a Nomogram to quantitatively predict the prognosis of KIRC patients. **(A)** Nomogram to quantitatively predict 1, 3, and 5-year overall survival in KIRC patients. **(B)** ROC curves for Nomogram, risk scores, and Other clinical features. **(C–E)** The calibration chart shows that the Nomogram has a pretty predictive ability.

In order to make our risk signature fully utilized in the clinical diagnosis and treatment of KIRC. We built a nomogram survival prediction map based on four independent clinical prognostic factors (age, grade, N, and stage) and risk score to quantitatively predict the 1, 3, and 5-year survival of KIRC patients (Figure 7A). By comparing the area under the ROC curve, we could see that our nomogram had good performance in the prediction of the prognosis of KIRC patients compared to other predictive indexes (Figure 7B). Subsequently, the calibration curves were employed to verify the prediction ability and accuracy of the nomogram, and the results showed that the nomogram could accurately achieve the prediction of the prognosis of patients with KIRC (Figure 7C–E), which also illustrated the value and potential of the nomogram in clinical application to achieve the prediction of the prognosis of patients with KIRC.

### Functional enrichment analysis based on risk signature

**FIGURE 8:**
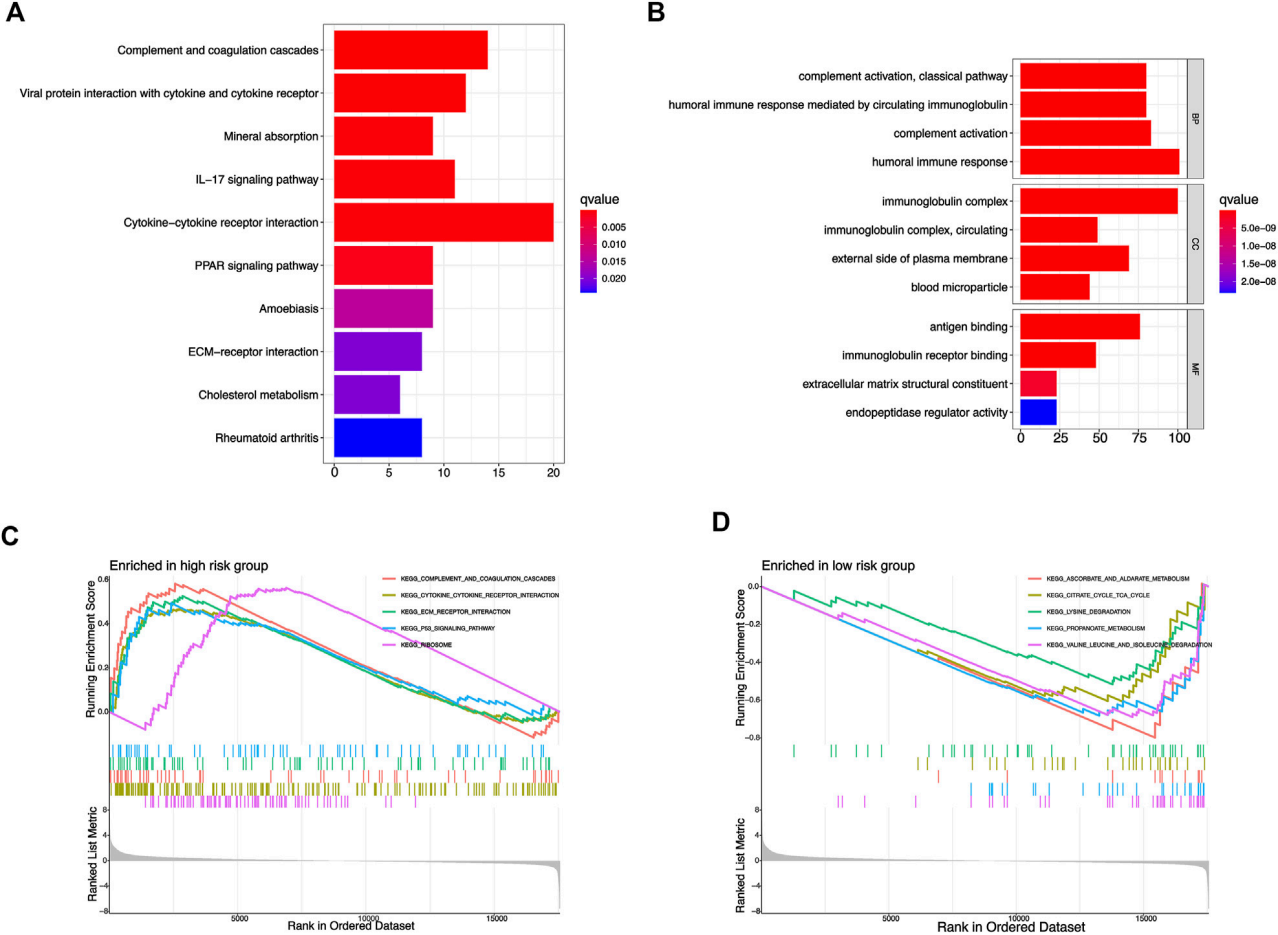
Functional enrichment analysis based on risk signature. **(A)** KEGG enrichment analysis of differential genes from risk signature. **(B)** GO enrichment analysis of differential genes from risk signature. **(C,D)** GSEA enrichment analysis of different risk groups.

Because the prognosis of KIRC patients in different risk groups was significantly different, we performed KEGG, GO, and GSEA enrichment analysis to preliminarily exploring the potential biological function differences between the two risk groups. We screened a total of 588 genes with differential expression from the risk_high_ and risk_low_ groups, and KEGG, GO enrichment analysis provided us with a biological understanding of the above genes (Supplementary Files S2). KEGG enrichment analysis suggested that the above genes were significantly associated with functional pathways such as IL 17 signaling pathway, Cytokine-cytokine receptor interaction, PPAR signaling pathway, etc (Figure 8A), and GO enrichment analysis suggested that the above genes were significantly related to biological behavior such as humoral immune response, immunoglobulin complex, immunoglobulin, etc (Figure 8B). In addition, we used GSEA software for the investigation of biological pathways that are enriched in risk_high_ and risk_low_ groups. With *p* < 0.05 as the standard, 41 biological pathways were defined enriched. The top 5 pathways enriched in risk_high_ groups were P53 signaling pathway, ECM receptor interaction, Complement, and coagulation cascades, Cytokine-cytokine receptor interaction, and Ribosome (Figure 8C). The top 5 pathways enriched in risk_low_ groups were: Ascorbate and aldarate metabolism, Citrate cycle tca cycle, Lysine degradation, Propanoate metabolism, Valine leucine and isoleucine degradation (Figure 8D). The above mechanisms might partly explain that why risk_high_ KIRC patients tend to have worse clinical outcomes than risk_low_ KIRC patients.

### The tumor immune microenvironment of KIRC patients was analyzed based on risk score

**FIGURE 9:**
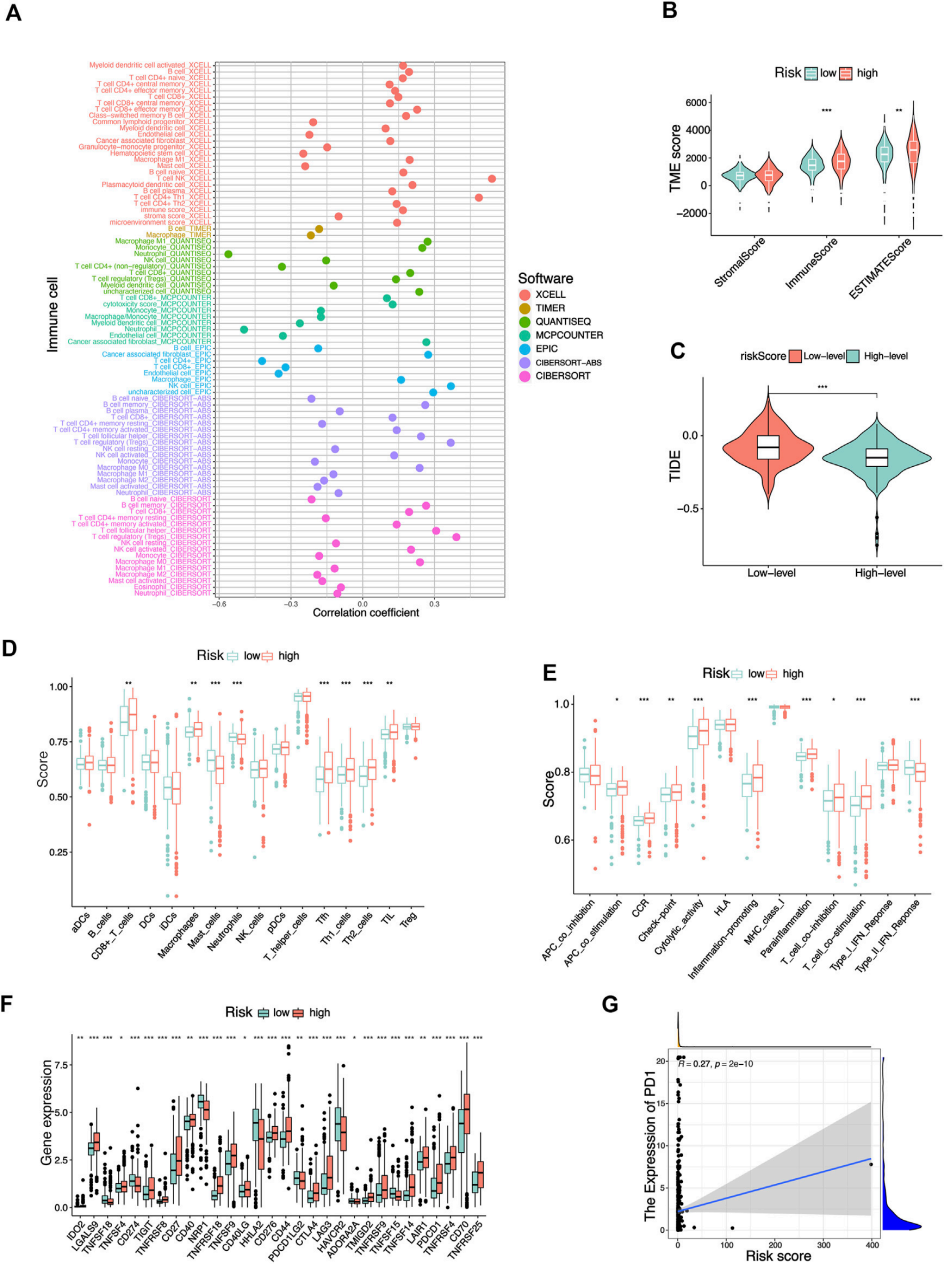
Potential effects of risk signatures on tumor immune microenvironment in KIRC patients. **(A)** The correlation between different immune cells and risk scores was analyzed based on multiple algorithms. **(B)** Differences in StromalScore, ImmuneScore, and ESTIMATEScore in KIRC patients with different risk groups (ESTIMATE algorithms). **(C)** Differences in immunotherapy sensitivity among different risk groups (TIDE algorithms). Differences in enrichment scores of 16 immune cells **(D)** and 13 immune-related pathways **(E)** in different risk groups. **(F)** Differential expression of common immune-checkpoints in different risk groups. **(G)** Correlation between risk scores and PD1 expression in KIRC patients. *p < 0.05, **p < 0.01,***p < 0.001.

Since the results of the previous functional analysis showed a close association with function and pathway of immunity, we further studied the status of immunization of KIRC patients between the risk_high_ and risk_low_ groups. First, a variety of algorithms were adopted to study the correlation between risk score and tumor immune cell infiltration, and there was a significant correlation between a variety of immunocytes and risk score (Figure 9A). Based on the ESTIMATE algorithm, we could conclude that the immune score of KIRC patients in the risk_high_ group is higher than that in the risk_low_ group (Figure 9B). Compared with risk_low_ KIRC patients, the risk_high_ KIRC patients had more active status of immunization and lower tumor purity. Then, we compared the differences of 16 types of immune cell enrichment scores and 13 types of immunization-associated function enrichment scores between the risk_high_ and risk_low_ groups (ssGSEA algorithm). Most immune cell and immunization-associated pathway enrichment scores were higher in the risk_high_ group, such as T cell co-stimulation, parainflammation, APC co-stimulation, Check-point, CD8^+^ T cell, Th1 cells, Th2 cells (Figure 9D,E). Thus it could be shown that immune activity was stimulated in patients with risk_high_ KIRC. The expression of some common immune checkpoint genes in both risk groups were also analyzed. Most of the immune checkpoints were expressed higher in the risk_high_ group (Figure 9F). For instance, *PD-1* (*PDCD1*) expression was higher in risk_high_ patients with KIRC compared with risk_low_ patients, and the expression of *PD1* increased as the risk score increased (Figure 9E), suggesting that *PD1* inhibitors may be more effective in high-risk score KIRC patients. In contrast, another common immunosuppressant *PDL1* (*CD274*) was higher in risk_low_ KIRC, meaning that risk_low_ KIRC patients might be more sensitive to treatment with *PDL1* inhibitors. Subsequently, we also compared the TIDE scores of the two risk groups (Figure 9C). The risk_low_ KIRC patients had higher TIDE scores and higher immune escape potential, suggesting that risk_low_ groups that received most immune checkpoint blockers had poor treatment effects. In general, risk_high_ KIRC patients had a more active immune state and were more likely to benefit from immunotherapy.

### Tumor mutation burden and prediction of potential drug sensitivity

**FIGURE 10:**
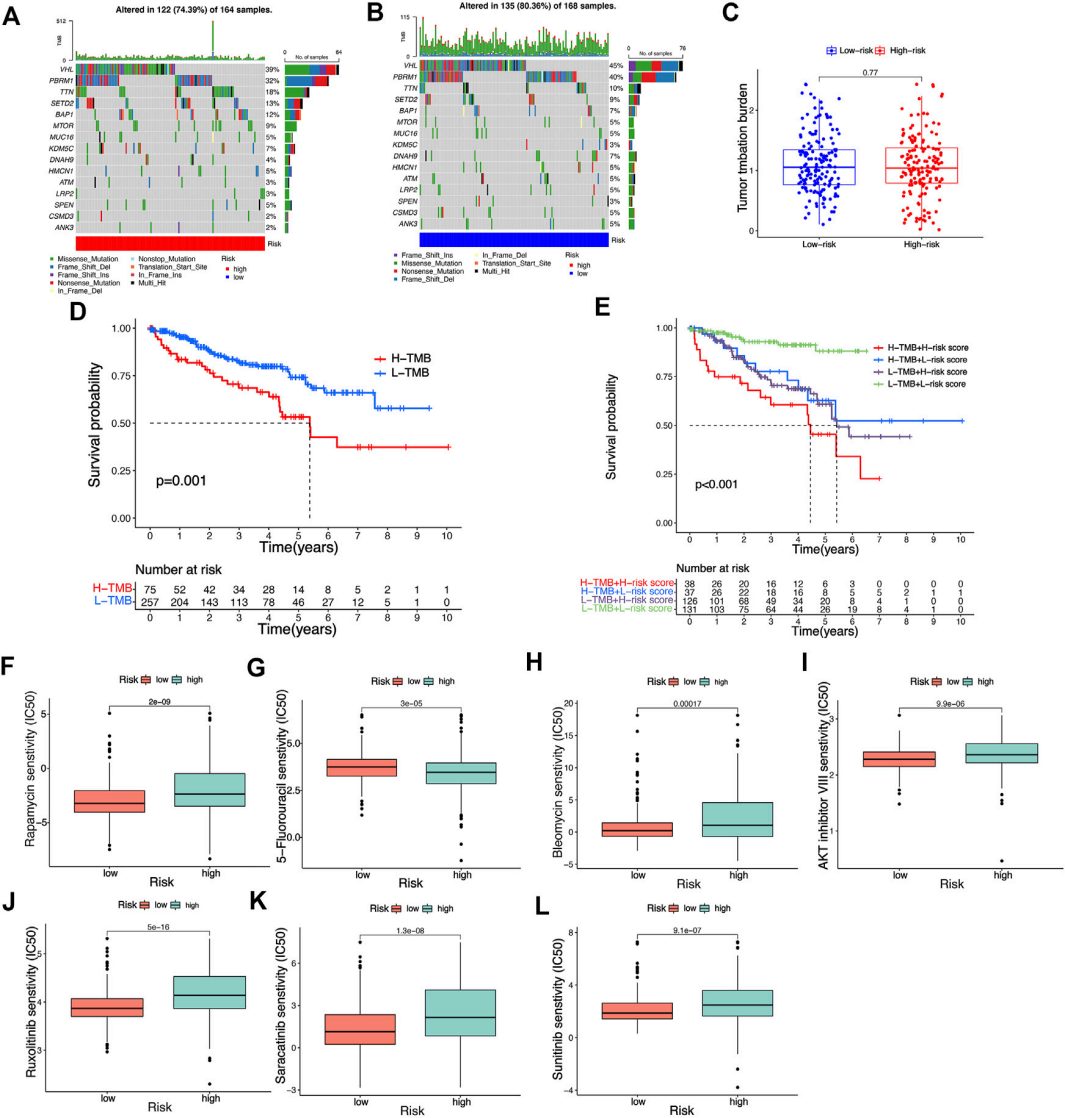
Analysis of tumor mutation burden and prediction of potential drug susceptibility in different risk groups. **(A,B)** The waterfall plot illustrates the type and frequency of tumor mutational burden in high-risk and low-risk KIRC patients. **(C)** Differences in tumor mutation burden scores among different risk groups. **(D,E)** K-M survival curves of KIRC patients with different risk scores and tumor mutation burden scores. **(F–L)** Differences in IC50 values of common drugs in different risk groups.

In accordance with the analysis result of somatic mutation data of KIRC patients in TCGA database, as shown in the waterfall diagram, 39.64% of risk_high_ KIRC patients had genetic mutation, while 38.68% of risk_low_ patients had such mutation (Figure 10A,B). TMB refers to the number of somatic non-synonymous mutations in the genomic region, which can indirectly reflect the ability and degree of neoantigen production of tumors and predict the efficacy of immunotherapy for a variety of tumors. TMB can be affected by many factors. KIRC patients with different clinical and biological characteristics have different TMB. Although the degree of TMB was higher in the low-risk group, this was not statistically significant. This may be due to the insufficient number of samples of KIRC patients who participated in the analysis of tumor mutational burden. Overall, these analyses provided a basis for risk signatures to predict the prognosis of KIRC patients and the effect of immunotherapy. (Figure 10C). In KIRC patients of all risk groups, the gene with the highest mutation frequency is *TNN*, and the most common mutation type is missense mutation. In accordance with the results of the K-M analysis, the OS rate of KIRC patients with high TMB is lower than that of KIRC patients with low TMB (Figure 10D). Next, the TMB and risk score of KIRC patients for survival analysis were integrated, and a conclusion was drawn that patients with high TMB and the high-risk score achieved the worst prognosis (Figure 10E), thus confirming the ability of our risk signature to predict the OS of KIRC patients. Although the difference in TMB score among different risk groups was not significant, it also revealed some potential mechanisms that might affect the clinical outcome of patients with KIRC. By comparing the IC50 values of some common drugs in different risk groups, we found that the risk score of KIRC patients could influence their sensitivity to drugs to a certain extent. As revealed by the results, the IC50 value of Rapamycin, Sunitinib, Bleomycin, AKT inhibitor VII, Ruxolitinib, 5-Fluorouracil, Saracatinibin risk_high_ groups was higher than that in risk_low_ groups, thus suggesting that risk_low_ groups may be more sensitive to the above drug treatment. (Figure 10F–L). The prediction of the efficacy of the above potential drugs for KIRC patients is beneficial to guiding the clinical drug treatment of KIRC patients. The above results also suggest that our risk signature in this study takes on certain significance for guiding drug therapy in patients with KIRC.

### Further studies on four LncRNAs associated with cuproptosis that constitute risk signature

**FIGURE 11:**
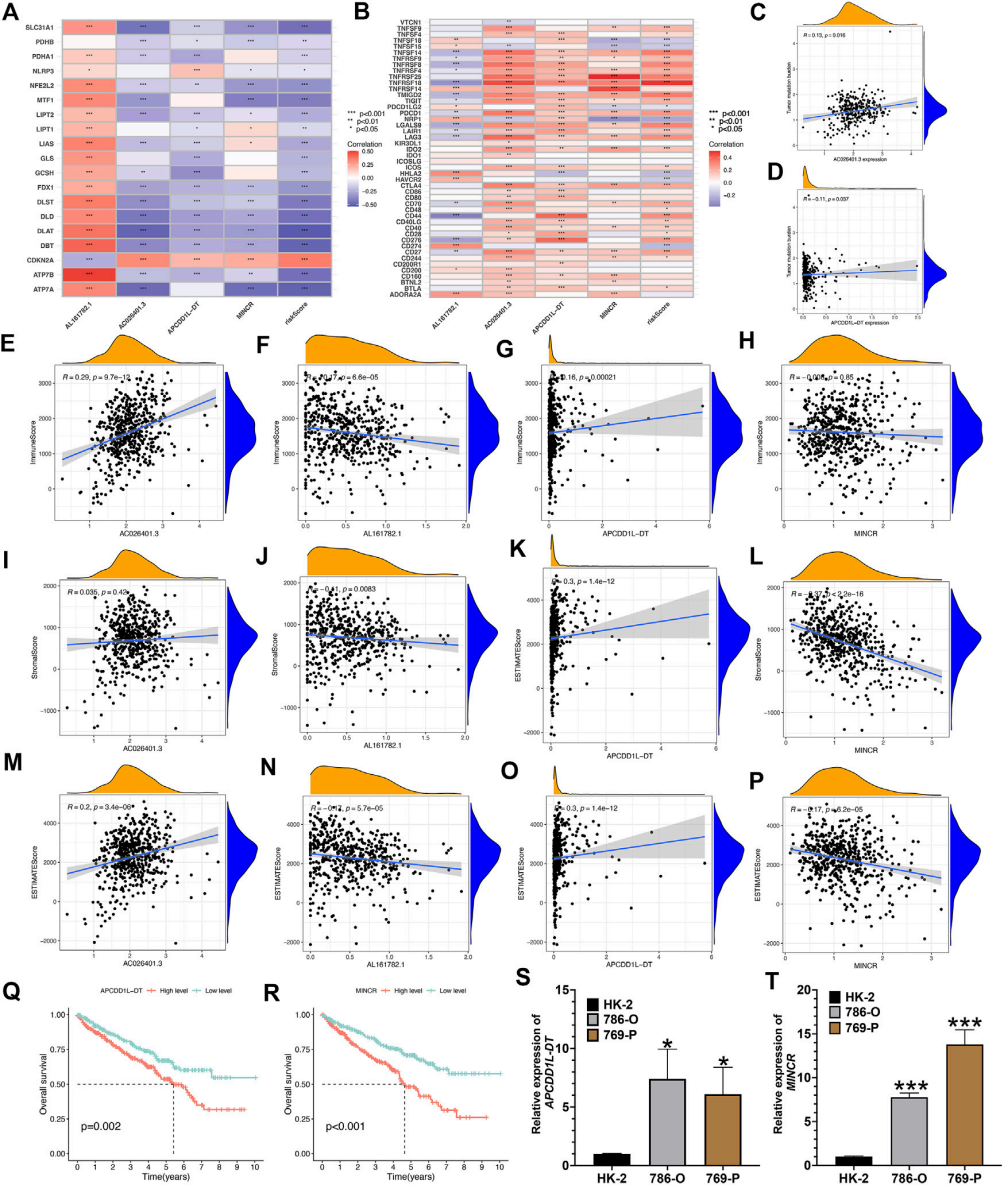
Comprehensive analysis of 4 risk cuproptosis-related lncRNAs. **(A)** Correlation between 4 risk LncRNAs and 19 cuproptosis-related genes. **(B)** Correlation between 4 risk LncRNAs and common immune-checkpoints. The scatter plot represents the correlation between the tumor mutation burden and the expression of AC026401.3 **(C)** and APCDD1L-DT **(D)**. **(E–P)** The scatter plots show the correlations between StromalScore, ImmuneScore, ESTIMATEScore, and the expression of the 4 risk lncRNAs. K-M survival curves analysis of APCDD1L-DT **(Q)** and MINCR **(R)**. Relative expression of APCDD1L-DT **(S)** and MINCR **(T)** in normal renal tubular epithelial cell line HK-2 and renal clear cell carcinoma cell line 786-O and 769-P.*p < 0.05, **p < 0.01,***p < 0.001.

Existing researches have suggested that the risk score of predictive signature is significantly associated with KIRC tumor immune microenvironment and TMB. Therefore, we further explore the biological characteristics of the 4 risk LncRNAs that make up the predictive signature from the perspective of tumor immunity and tumor mutation. As depicted in Figure 11A, the 4 risk LncRNAs were significantly associated with most genes associated with cuproptosis. Next, we also analyzed the correlation of common immune checkpoint genes and 4 risk LncRNAs (Figure 11B). It could be seen that common immune checkpoint PD1 and PDL1 are not only related to risk scores, but also significantly related to 4 risk LncRNAs. The correlation scatters plot showed the correlation between risk LncRNAs and TMB (Figure 11C,D). Based on the results of the ESTIMATE algorithm, it was found that the expression of 4 risk LncRNAs was potentially related to the microenvironment of tumor of KIRC (Figure 11E–P). Existing researches have suggested that *MINCR* and *APCDD1L-DT*, are significantly associated with the occurrence and growth of various tumors, such that the focus of this study was placed on the biological characteristics of two risk LncRNAs in KIRC. First, the K-M survival analysis curve of 530 KIRC patients indicated that the expression of the above two risk LncRNAs was significantly related to the OS rate of KIRC patients (Figure 11Q,R). Subsequently, the relative expression of LncRNA of *MINCR* and *APCDD1L-DT* was detected through qRT-PCR in HK-2, 786-O, and 769-P cells. The results indicated that the relative expression level of *MINCR* and *APCDD1L-DT* was higher in 786-O and 769-P tumor cells than that in HK-2 cells, which were normal renal tubular epithelial cells (Figure 11S,T). In general, the experimental results of this study indicated the accuracy of our risk signature to a certain extent.

## Discussion

KIRC is one of the most common malignant tumors of the urinary system, which has the characteristics of high recurrence rate, high risk of metastasis, and poor prognosis, especially for advanced KIRC patients, whose prognosis is often very unsatisfactory (Umberto et al., 2019). Considerable previous research over the past few years suggested that LncRNAs take critical significance in regulating the occurrence and growth of KIRC. At present, many prognosis prediction models based on LncRNA have been applied to KIRC (Xinfang et al., 2021). Xing’s research developed s risk assessment model by ferroptosis-related LncRNAs could accurately achieve the prediction of the prognosis of KIRC patients (Xiao-Liang et al., 2021). Tang’s team defined a signature of ferroptosis-related LncRNAs that could improve the prognosis prediction in papillary renal cell carcinoma (Xinfang et al., 2022). Yu et al. found that the prognosis model based on m6A-related LncRNAs can provide powerful help for the prognosis evaluation of KIRC patients (JunJie et al., 2021). Professor Sun’s team pointed out that immunization-associated LncRNAs can not only help achieve the prediction of the prognosis of KIRC patients but also become a potential immunotherapy target for KIRC patients (Zhuolun et al., 2020). Cuproptosis—as a newly discovered regulation mode of cell death, some studies on cuproptosis in KIRC have also been reported (Ganghua et al., 2022). For example, FDX1, a key gene of cuproptosis, which had been proved to have a certain effect on the proliferation of KIRC cells, in addition, the presence of cuproptosis was demonstrated in KIRC cells (Ganghua et al., 2022). Cuproptosis could bring more options for immunotherapy and targeted therapy for KIRC patients. However, the current research on CRLs in KIRC is blank, and there is no KIRC prognosis prediction model based on CRLs. In this study, we first applied CRLs to KIRC. Cox and LASSO regression analyses were carried out for constructing and verifying the CRLs prognosis signature, which had good predictive performance. The potential correlation between prognosis signature and microenvironment of KIRC, immune checkpoint, and TMB were also systematically studied. The above results are expected to help the clinical diagnosis and treatment of KIRC.

We downloaded 539 KIRC tissue specimens and 72 normal kidney tissue specimens and their corresponding clinical information from the TCGA database. Genes associated with cuproptosis were obtained from previous literature studies. The CRLs were determined by spearman correlation analysis. Then, based on Cox and LASOO regression analysis, a prognostic risk signature composed of 4 risk CRLs was built. The result suggests that the OS、PFS rate of KIRC patients with high-risk scores is less than that of KIRC patients with low-risk scores. The ROC curve showed that the AUC values of 1, 3, and 5 years in the training group were 0.828, 0.780, and 0.794, respectively, suggesting that the prognosis signature built by CRLs had high accuracy and reliability. At the same time, we also consulted other published LncRNA prognostic models. By comparing the AUC values of 1-year risk models, we found that our prognostic model has better predictive performance than other published models. Furthermore, the clinical factors and risk score were integrated to build a nomogram, thus making the developed prognosis signature more applicable to clinical trials.

As the enrichment results of KEGG, GO and GSEA all show that many enrichment pathways are showed significantly associated with immune activities, we discussed the correlation between risk score and KIRC tumor immune microenvironment. The immune score and ESTIMATE score of the risk_high_ KIRC patients were higher than those of the low-risk. The change of tumor immune microenvironment could promote the proliferation, migration, and invasion of KIRC, which explains to a certain extent that there were significant survival differences among KIRC patients in different risk groups. In addition, based on various algorithms, we analyzed the differences in immune activities of KIRC patients among different risk groups. It could be seen that most immunocytes and immunization-associated functions have higher enrichment scores in risk_high_ KIRC patients, and risk_high_ KIRC patients have more frequent immune activities. In recent years, immunotherapy has been widely used in patients with KIRC, especially in patients with advanced KIRC. For instance, PD1 and PDL1 inhibitors can improve the survival rate of some patients with advanced KIRC to a cerntai extent, but unfortunately, not all patients with KIRC can benefit from them. Accordingly, we try to provide individualized immunotherapy for KIRC patients based on our risk signature. We compared the expression of common immune checkpoint genes in different risk groups and suggested that most immune checkpoints have higher expression in risk_high_ groups, such as PD1, CD27, and CD40, which indicates that risk_high_ KIRC patients may be more sensitive to the treatment of the above immune checkpoint inhibitors, and can get better therapeutic effects from them. In addition, compared with the low-risk KIRC patients, the TIDE score of the risk_high_ KIRC patients was lower, indicating that the risk_high_ KIRC patients have lower immune escape potential. It also showed that risk_high_ KIRC patients can benefit from ICB treatment. The above immune analysis results revealed that risk_high_ KIRC patients might gain more benefits from immunotherapy. Lastly, we predicted the sensitivity of some potential therapeutic drugs based on the risk signature, which could help the clinical drug treatment of KIRC patients. For example, Rapamycin, Sunitinib, Bleomycin, AKT inhibitor VII, Ruxolitinib, 5-Fluorouracil, Saracatinibin. According to the latest clinical guidelines, targeted therapies for renal cancer are mainly divided into two categories: tyrosine kinase inhibitors (TKI) and m-TOR inhibitors. TKI drugs are important means to treat metastatic renal cancer, and currently, commonly used drugs include sunitinib, ruxolitinib, sorafenib, and axitinib. m-TOR inhibitors, which target m-TOR and related signaling pathways, can control the proliferation and angiogenesis of tumor cells, so as to control the tumor. Commonly used drugs include rapamycin and everolimus, which are mainly used as second-line drugs for advanced renal cancer patients who have failed TKI treatment. Our study compared the IC50 values of these drugs in different risk populations to predict the sensitivity of different risk populations to these targeted therapies, so as to guide clinical application.

Most research has suggested that LncRNA takes on critical significance in developing some common malignant tumors over the past few years, so the role played by four risk LncRNAs making up the prognosis signature was investigated in depth. There has been little research about *LncRNA AL161782.1*. Several research has highlighted that *LncRNA AC026401.3* plays a role in building the model for the prediction of the prognosis of hepatocellular carcinoma and renal carcinoma. *LncRNA AC026401.3* takes on significance in the prognosis of patients with hepatocellular carcinoma and renal carcinoma to a certain extent, and *LncRNA APCDD1L-DT* can serve as a marker of the prognosis of lung squamous cell carcinoma (Honghao et al., 2021; Rongjiong et al., 2021; Min et al., 2022). A considerable number of studies suggested that the expression of *LncRNA MINCR* is capable of affecting a wide variety of malignant tumors’ development and occurrence. For instance, Li’s experimental research proves that *LncRNA MINCR* can regulate the *miR-876-5p*/*GSPT1* axis to aggravate the progression of glioma (Zheng et al., 2020). Yu et al.’ s research shows that *LncRNA MINCR* has high expression in colon cancer tissues and cells, and promotes the proliferation and migration of colon cancer by regulating *miR-708-5p* (Yang et al., 2020). However, the research on the above four risk LncRNAs in KIRC is still very blank, especially *LncRNA MINCR*, many studies have shown that it can play a role as an oncogene in the development of various tumors. The qRT-PCR experiment also demonstrated that *MINCR* and *APCDD1L-DT* have high expression in KIRC cells. Furthermore, this study also shows that the expression of the above-risk LncRNAs is significantly associated with immune activities. The four risk LncRNAs, especially *MINCR*, have the potential to become a new target of KIRC immunotherapy. We also expect that the further research results of the above LncRNAs can guide the clinical diagnosis and treatment of KIRC.

In general, this study has certain clinical values and limitations. First of all, we studied the genes associated with cuproptosis for the first time and built a prognosis model based on CRLs, which can accurately achieve the prediction of the prognosis of KIRC patients, and its predictive performance is better than some published models for the prediction of the prognosis of KIRC patients. Second, we also systematically analyzed the correlation between the CRLs prognosis signature and tumor immune microenvironment, which provided a new idea for guiding the immunotherapy of KIRC. Thirdly, we also showed a novel direction for the drug treatment of KIRC. Lastly, we proposed that *LncRNA MINCR* has great potential to become a new target of KIRC immunotherapy. However, this study also has some limitations. First, our signature was only verified internally, and the expression of risk LncRNA was simply verified by qRT-PCR. No suitable external dataset was identified in the published database to further evaluate the reliability of our signature. Second, we lack clinical follow-up data to prove the value of our prognostic model. Lastly, in-depth *in vivo* and *in vivo* experiments should be performed to verify the conclusion of this study, especially to verify the role of *MINRC i*n the development of KIRC. Although there are several defects, the CRLs prognosis signature can accurately achieve the prediction of the prognosis of KIRC patients, which is initially found in this study. Therefore, this study has a great application prospect in clinical practice.

## Conclusion

The team of this study built a risk signature based on cuproptosis-related LncRNAs, which can precisely achieve the prediction of KIRC patient’s prognosis. In accordance with the risk signature, we evaluated the role played by cuproptosis-related LncRNAs in the immune microenvironment of KIRC tumors and the possible potential regulatory mechanism, which is conducive to guiding the individualized treatment of KIRC. In addition, four identified risk lncRNA (especially *MINCR*) can be novel targets for immunotherapy of KIRC patients (Table 2).

**TABLE 2:** Multivariate Cox regression analysis of 4 risk lncRNAs.

Risk LncRNAs	Coeficient	HR	HR.95L	HR.95H
*AL161782.1*	−1.1512239	0.31624946	0.13522153	0.73962866
*AC026401.3*	0.47111037	1.60177177	0.99208715	2.58613652
*APCDD1L-DT*	0.6788922	1.97169228	1.35943561	2.85969446
*MINCR*	0.46866756	1.59786372	1.05959843	2.40956233

## Data Availability

The datasets presented in this study can be found in online repositories. The names of the repository/repositories and accession number(s) can be found in the article/Supplementary Material.

## Abbreviations

RCC, renal cell carcinoma; KIRC, kidney renal clear cell carcinoma; LncRNAs, long non-coding RNAs; CRLs, cuproptosis-related LncRNAs; TCGA, the cancer genome atlas; CNV, copy number variation; TMB, tumor mutation burden; TIDE, tumor immune dysfunction and exclusion; FDR, false discovery rate; OS, overall survival; PFS, progression-free survival; ROC, receiver operating characteristic; AUC, area under the ROC curve; K-M, kaplan-meier; GO, gene ontology; KEGG, kyoto encyclopedia of genes and genomes; GSEA, gene set enrichment analysis; PCA, principal component analysis; FC, fold change.
